# The single-leg heel raise does not predict maximal plantar flexion strength in healthy males and females

**DOI:** 10.1371/journal.pone.0253276

**Published:** 2021-08-20

**Authors:** Lauren K. Sara, Savannah B. Gutsch, Sandra K. Hunter

**Affiliations:** Department of Physical Therapy, Marquette University, Milwaukee, Wisconsin, United States of America; University of Tennessee Health Science Center College of Graduate Health Sciences, UNITED STATES

## Abstract

**Introduction:**

The single-leg heel raise test (SLHR) is commonly used in clinical settings to approximate plantar flexor strength, yet this is neither validated nor supported physiologically. The purposes of this study were to: determine (1) associations between SLHR repetitions, maximal plantar flexor strength, and reductions in strength; and (2) whether sex differences exist in performance of the SLHR.

**Methods:**

Twenty-eight young, healthy participants (14 males,14 females, 19–30 years) performed repeated single-leg heel raises to task failure. Pre- and post-task measures included maximal voluntary isometric contractions (MVIC), and voluntary activation and contractile properties of the plantar flexor muscles, assessed using peripheral electrical stimulation of the tibial nerve. Surface electromyography was recorded for the medial and lateral gastrocnemius, soleus, and anterior tibialis muscles.

**Results:**

The SLHR resulted in 20.5% reductions in MVIC torque (p<0.001). However, the number of SLHR repetitions was not correlated with either the baseline MVIC (maximal strength; p = 0.979) or the reduction in MVIC following the SLHR (p = 0.23). There were no sex differences in either the number of SLHR repetitions (p = 0.14), baseline MVIC torque (p = 0.198), or the reduction of MVIC (p = 0.14). MVIC decline was positively associated with the reduction in voluntary activation (r = 0.841, p<0.001), but was not associated with the change in twitch amplitude (p = 0.597).

**Conclusions:**

The SLHR was similar in young males and females yet was a poor predictor of maximal plantar flexor strength but evaluates performance fatigability of the lower extremity specific to dynamic contractions. The reduction in maximal strength at task failure was explained by reduced neural drive to the plantar flexor muscles in both males and females.

**Impact statement:**

SLHR performance is not a clinical assessment of plantar flexor strength but assesses dynamic lower extremity fatigability that is similar in males and females. Alternate clinical measures for maximal plantar flexion strength need to be developed.

## Introduction

The single-leg heel raise test (SLHR) is commonly used in clinical practice as a manual muscle test of plantar flexion strength. It is a dynamic task, assigning strength grades based on repetitions completed before task failure [1]. This approach differs from the traditional manual muscle testing approach, which involves evaluation of maximal *isometric* strength against resistance provided by an examiner [2,3]. While the SLHR is widely adopted in the clinic, there are several limitations for using it as a measure of antigravity plantar flexion strength. First, the repetitions required for a maximal strength grade vary widely between sources, and healthy populations vary between completing zero and 120 repetitions [2,4]. Second, task performance in the SLHR cannot be isolated to plantar flexion across the ankle. To maintain upright posture during the task, the body must appropriately respond to perturbations in three planes of motion. Lastly, and perhaps most importantly, the assertion that the SLHR correlates well with maximal isometric strength is not validated [5], nor supported physiologically. In fact, the number of repetitions completed before task failure is a measure of performance fatigability (endurance), not strength [6,7].

Performance fatigability is an acute, activity-induced decline in an objective measure of motor performance over time [8]. In laboratory and clinical settings, fatigability is often quantified as an exercise-induced reduction in the maximal force or power of the exercising limb [6,9,10]. For submaximal intensity tasks with a constant load—such as body weight during the SLHR—fatigability can be quantified as the time or number of repetitions completed before failure [7]. Fatigability and the contributing mechanisms (e.g. neural and muscular mechanisms) vary between muscle groups, between tasks (e.g. dynamic vs isometric tasks), and between populations [10,11], including between males and females [12]. The SLHR purports to measure plantar flexion strength during a dynamic task. However, strength and fatigability are often not associated, possibly because the metabolic demands are quite different; in fact, in some populations, these have an inverse relationship, whereby weaker individuals exhibit a greater task duration [13]. There is no information on 1) the relationship between maximal strength and the clinical test of SLHR; 2) the magnitude of fatigability that occurs during the SLHR; and 3) whether there are differences between males and females, as is often observed for other tasks [12,14,15].

Sex differences in fatigability exist across many muscle groups and tasks, both in terms of the magnitude of fatigability and the neural and muscular mechanisms contributing to fatigability [12]. Males, who are often stronger, are typically more fatigable than females [12]. This is especially true during isometric tasks and is more variable with dynamic fatiguing tasks [12,16]. If sex differences exist in SLHR performance, the criteria for muscle grades could differ between populations, such as between males and females. Importantly, knowledge of these will help guide clinicians when executing and evaluating the SLHR in clinic.

The purpose of this study was to determine: 1) associations between SLHR repetitions and measures of maximal plantar flexion strength, assessed as baseline maximal voluntary isometric contraction (MVIC); 2) associations between SLHR repetitions and the reduction in MVIC following the SLHR; and 3) whether sex differences exist in performance of the SLHR. We *hypothesized* that: 1) the number of SLHR repetitions would be poorly correlated with a measure of baseline maximal plantar flexion strength; 2) the number of SLHR repetitions would be associated with a measure of plantar flexor fatigability (measured as the reduction in maximal plantar flexor strength); and 3) males would demonstrate greater fatigability than females (measured as SLHR repetitions and the reduction in MVIC). To understand the contribution of neural mechanisms to fatigability of the plantar flexor muscles, the reduction in voluntary activation (central fatigue) was assessed with the interpolated twitch technique during MVICs before and after the SLHR [9,14]. Muscular mechanisms were assessed as the change in contractile function and represented as the change in electrically-evoked involuntary contractions of the plantar flexor muscles [9].

## Methods

### Participants

Thirty healthy, young adults (15 males and 15 females, 19–30 years) volunteered to participate in the study. Exclusion criteria included known cardiovascular disease, neurological disease, and a history of musculoskeletal injury to the right lower extremity. Informed consent was obtained from all participants prior to participation in the study. The study protocol was approved by the Marquette University Institutional Review Board and in compliance with the Declaration of Helsinki.

**Fig 1:**
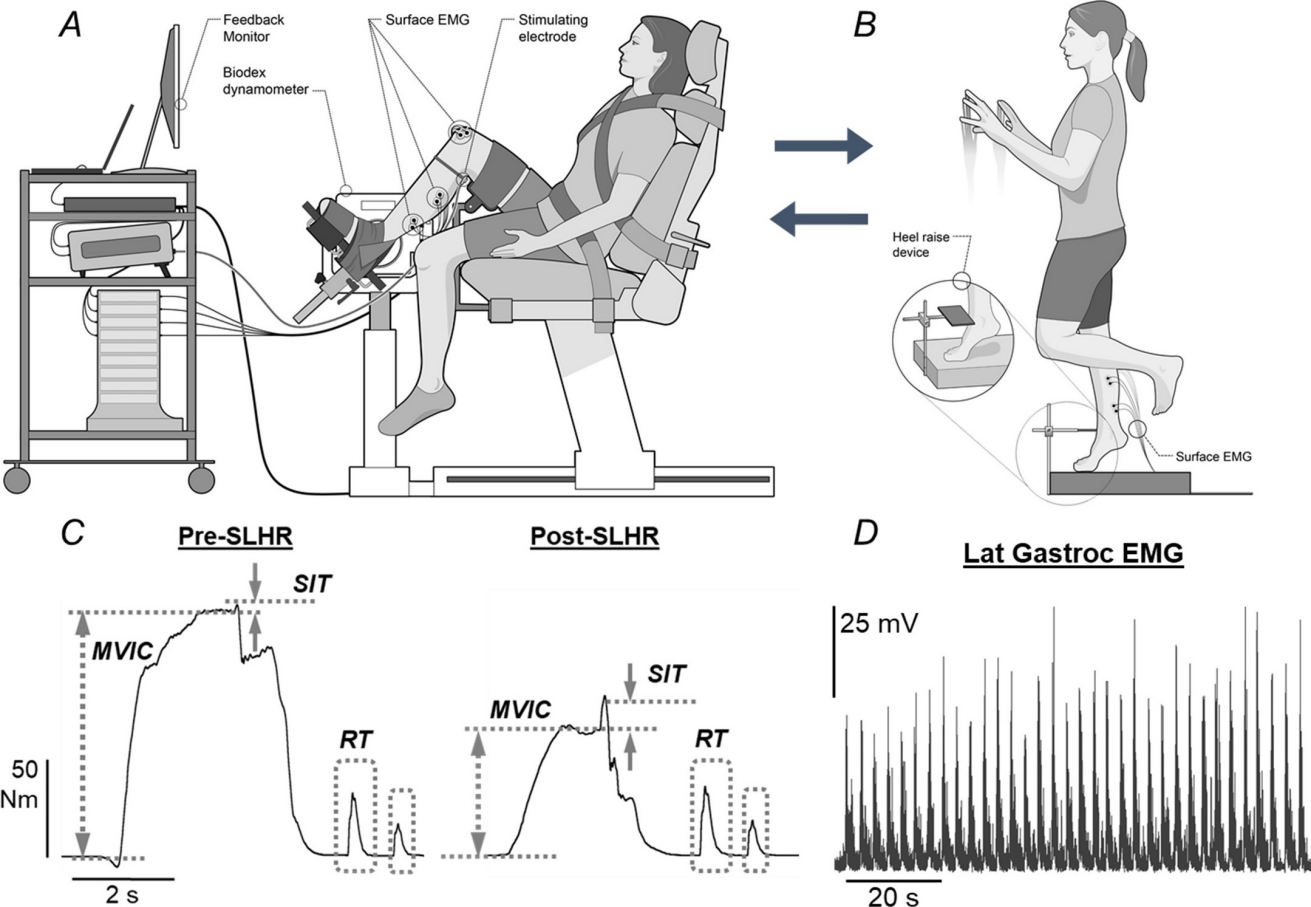
Experimental setup, study protocol, and raw data. ***A*:** Experimental setup of a participant in the Biodex System3 Pro dynamometer used for baseline and post-SLHR task measurements. Participants were given visual feedback on a monitor. Surface electromyography (EMG) was recorded for the medial and lateral gastrocnemius, soleus, and anterior tibialis muscles. Electrical stimulation was positioned over the tibial nerve. ***B*:** Experimental setup for the SLHR task. Participants stood on a custom-made heel raise device (see inset). Surface EMG was similarly recorded for the duration of the SLHR task. ***C*:** Representative torque data from a female participant, before and after the SLHR task. Measurements included: Maximal voluntary isometric contraction (MVIC), superimposed twitch (SIT), and resting twitch (RT). The MVIC decreased and SIT increased following the SLHR task, leading to reduced voluntary activation. ***D*:** Root-mean-squared (RMS) EMG data recorded during the SLHR task. The representative data is from the lateral gastrocnemius of the female participant from *C* and shows an increase in EMG amplitude.

All testing took place during one session by the same researcher and was performed on the right lower extremity. The study involved measures of strength (MVIC), voluntary activation and contractile properties of the plantar flexor muscles while seated in a Biodex dynamometer (Biodex System 3 Pro; Biodex Medical; Shirley, NY) before and after a single-leg heel raise task (SLHR) performed to failure. The SLHR was performed in the upright standing position on a custom-made heel raise device placed adjacent to the Biodex dynamometer (Fig 1B). Physical activity data were calculated based on responses to a 12-month self-report physical activity questionnaire, the Modifiable Activity Questionnaire (MAQ) [17].

### Experimental setup

Each participant was seated in a Biodex dynamometer to quantify forces during the MVIC and assess voluntary activation and contractile properties of the right plantar flexor muscles. The right hip and knee were flexed to 90 degrees, the thigh resting on a padded thigh support, and the foot resting on a foot plate affixed to the dynamometer (Fig 1A). To minimize extraneous movement and isolate exercise to the right ankle joint, straps were placed across the waist, chest, and right thigh. Two straps were used around the ankle and one around the forefoot to maintain the plantar surface of the foot in contact with the foot plate. The right ankle was placed in a neutral position, with the foot perpendicular to the shank.

### Experimental protocol

#### Baseline measures

Participants performed MVICs of the plantar flexors with two-minutes rest between trials. Each participant performed at least two trials, repeating these until no further torque increases resulted (determined as two MVICs within 5% of each other).

The right tibial nerve was stimulated using a constant-current, variable high-voltage stimulator (DS7AH; Digitimer Ltd; Hertforshire, UK) to evoke contractions to assess contractile properties at rest and during MVICs (voluntary activation). Stimulation was applied to the right tibial nerve using a bar electrode at the medial popliteal space distal to the sciatic nerve bifurcation. Single square-wave pulses of 100μs duration (400 V, 100 Hz) were delivered with a stimulation intensity initiated at 50mA and gradually increased until a plateau was reached, such that further increases in stimulation intensity resulted in no additional increases in evoked torque [14,18]. The plateau intensity was increased further by 10%. This stimulation intensity was used during a single pulse stimulation and double pulse of stimulation (at 100Hz) to enable assessment of contractile properties and voluntary activation, respectively, of the plantar flexor muscles.

MVICs were repeated with the addition of both a superimposed doublet stimulation (SIT, superimposed twitch) followed by a resting (potentiated) doublet stimulation (RT, resting twitch) of the tibial nerve ~2 seconds after the MVIC (Fig 1C). Doublet stimulations were used for the SIT, as this has been shown to be more sensitive [19,20]. The forces during the SIT and RT were used in the analysis of voluntary activation and resting twitch torque (see Data Analysis). Each participant performed four MVICs with two minutes of rest between each trial.

#### Single-leg heel raise task

The SLHR involved repeated heel raises of the right leg while in upright standing. The SLHR task was performed using a custom-made heel raise device (Fig 1B) that included a horizontal plate affixed above a standing surface by an upright support bar. The horizontal plate served as a visual and tactile target during the SLHR and was adjustable in both the vertical and anteroposterior dimensions. The position of the horizontal plate was selected as the maximum height achieved during a single-leg heel raise for each subject, adjusted to the dorsal ankle crease at end-range during a single-leg heel raise.

Participants were familiarized and provided instruction about on performance of the SLHR prior to the baseline measures. This included the importance of contacting the horizontal target plate at each repetition and to demonstrate slow, controlled lowering during the eccentric phase of the task. Instability as a cofounding variable was minimized by allowing use of light fingertip support in the horizontal plane.

The SLHR was standardized to a pace of one heel raise every three seconds. This was achieved using a 60 beats-per-minute audible metronome and verbal cueing of “up, down, rest” to signify three-second epochs wherein participants took one second each to raise onto their toes, lower down, then rest. Verbal encouragement was provided and the SLHR was performed until task failure, defined as two consecutive missed contacts with the horizontal target plate. Participants were blinded to the failure criteria.

#### Post SLHR task measurements

Upon task failure, the participant was returned immediately to the Biodex for post-task measurements of MVIC with a SIT followed by resting twitches two seconds post MVIC. These measurements began at 45 ± 8 sec and were repeated at 1, 3, 5, 7, 9, and 11 mins post-SLHR.

#### Electromyography (EMG)

EMG activity of the medial gastrocnemius, lateral gastrocnemius, soleus, and tibialis anterior was recorded using bipolar EMG electrodes (Ag–AgCl, 8-mm diameter; 20 mm inter-electrode distance; Natus Medical Inc) located over the muscle bellies in accordance with recommendations by the SENIAM project [21]. In brief, electrodes were placed midway between the anatomical origin and myotendinous junction of each muscle, in alignment with the fiber direction of the respective muscle. Data were amplified (4000 Hz; Coulbourn Instruments, Allentown, PA), digitized (Power1401, Cambridge Electronic Design Limited, Cambridge, UK) and stored online using Spike2 software (Cambridge Electronic Design Limited, Cambridge, UK).

#### Data and statistical analyses

Thirty people volunteered to participate in this study. The data from one female and one male were removed because their MVIC torque and voluntary activation values were more than 3 standard deviations below the group means. Thus, 14 females (19–30 yrs, 21.1±2.9 yrs) and 14 males (19–26 yrs, 21.5±1.8 yrs) are included in the subsequent data and statistical analyses.

For all analyses of strength, contractile properties, and voluntary activation, “baseline” refers to the average of values that occurred during the two baseline MVICs that yielded the greatest torque amplitudes. “Post-task” refers to the first MVIC following the SLHR.

MVIC was measured as the average of a 0.5-second window surrounding the maximum torque. Voluntary activation was calculated as the ratio of the superimposed to the resting doublet twitch [9]: (1-SIT/RT)*100. The resting twitch refers to the torque amplitude of the electrically-evoked potentiated resting doublet stimulation that occurred immediately after an MVIC.

Muscle activity was quantified as the root-mean-square (RMS) value of the electromyography (EMG) signals for each the medial and lateral gastrocnemius, soleus, and tibialis anterior. Maximal baseline EMG was measured over the same 0.5-second interval as the MVIC, and this was used to normalize EMG data from the SLHR. For the SLHR, EMG data was quantified as the RMS value over a 0.5-second interval surrounding the maximal EMG activity recorded during each SLHR repetition.

Independent samples *t*-tests were used to compare physical characteristics, physical activity levels, number of SLHR repetitions completed, and baseline measures of strength, muscle properties and voluntary activation between males and females. Repeated-measures ANOVAs were used to evaluate changes in MVIC torque, electrically-evoked resting twitch amplitude, and voluntary activation from baseline to immediately post-SLHR, using time as the within-subjects factor and sex as the between-subject factor. Similarly, repeated-measures ANOVAs were used to evaluate changes in EMG activity from start to end of the SLHR task, using time as the within-subjects factor and sex as the between-subjects factor. For all ANOVAs, Greenhouse-Geisser corrections were used whenever the assumption of sphericity was violated. Mediation analyses were utilized to evaluate the impact of body weight on the relationships between sex and the outcome variables (Field-Fote 2019).

Pearson correlation was used to evaluate associations between SLHR repetitions, baseline dependent variables, and changes in dependent variables from pre- to post-task. Normality was assumed for all variables based on histograms and Q-Q plots. Significance was determined *a priori* at *p* < 0.05. Data are reported as mean ± standard deviation (SD) in the text and displayed as mean ± standard error of the mean (SEM) in the figures. All analyses were performed in IBM Statistical Package for Social Sciences (SPSS, V26).

## Results

Males and females were similar in both BMI and in self-reported physical activity levels, while males had more mass and were taller (see Table 1).

**Table 1:** Participant demographics & baseline characteristics.

Variable	Units	Males	Females	*p*-value
**N**		14	14	
**Age**	yrs	21.5±1.8	21.1±2.9	0.58
*Height	m	1.81±0.08	1.66±0.07	<0.001
*Weight	kg	79.4±10.3	64.0±10.8	0.001
**BMI**	kg·m−²	24.3±2.3	23.2±3.1	0.27
**Physical Activity**	met·hr·wk−¹	66.6±26.6	59.5±35.0	0.55
**MVIC**	Nm	148.8±36.3	128.1±34.1	0.20
**RT**	Nm	32.6±9.9	25.7±8.5	0.06
**VA**	%	91.5±8.2	90.9±10.2	0.87
**SLHR**	reps	32.6±6.9	39.4±14.8	0.14

* *p*<0.05;

** p<0.01.

BMI, body mass index, MVIC, maximal voluntary isometric contraction; RT, potentiated resting twitch amplitude; VA, voluntary activation; SLHR, single leg heel raise.

### SLHR and MVIC

**Fig 2:**
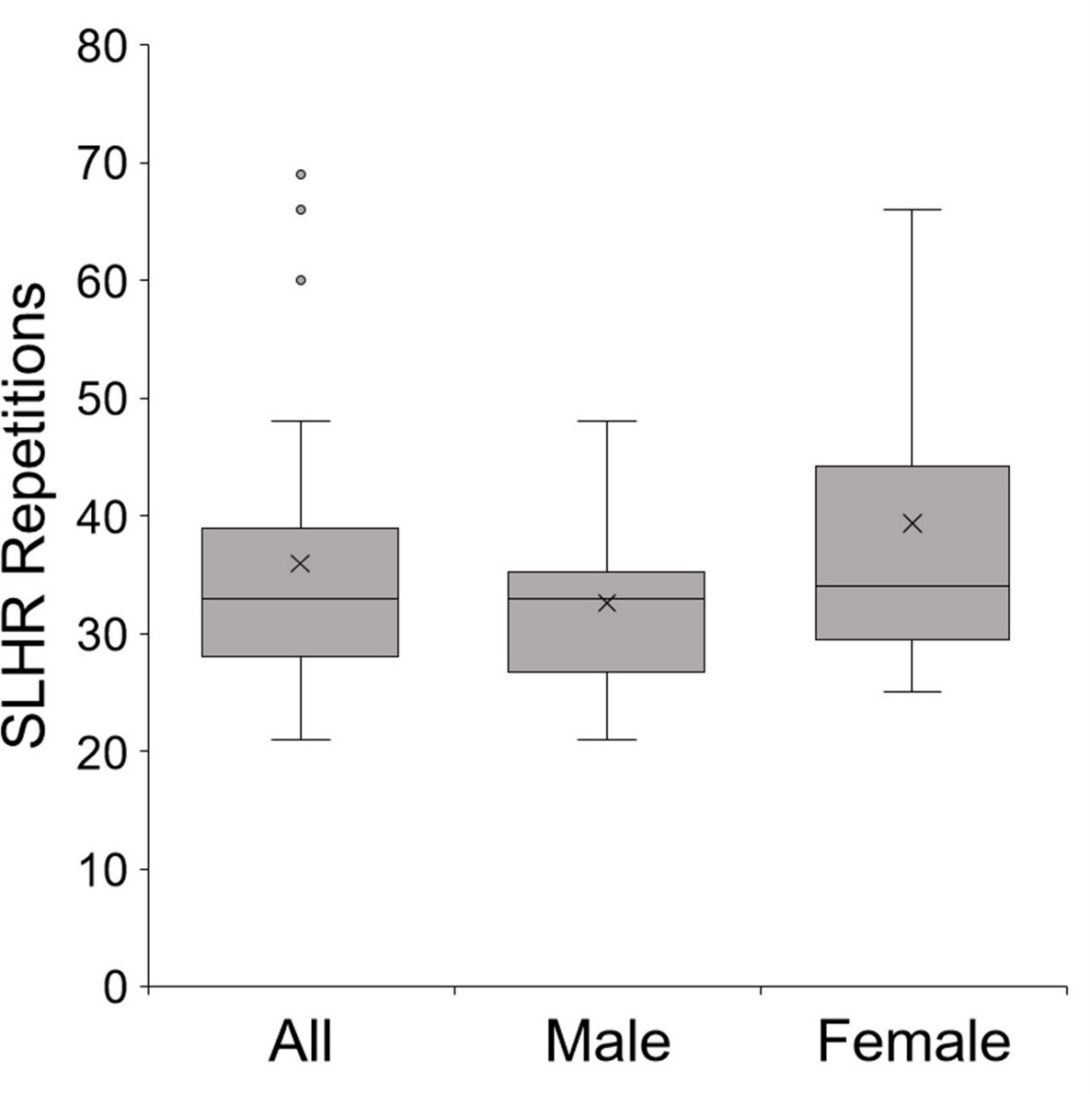
Box-and-whisker plot of single-leg heel raise (SLHR) repetitions performed by males and females. The middle line represents the median values, while the “x” represents the mean values. The left-hand box shows the pooled results for males and females. There were no sex differences in number of SLHR repetitions completed at time of task failure (p = 0.137).

**Fig 3:**
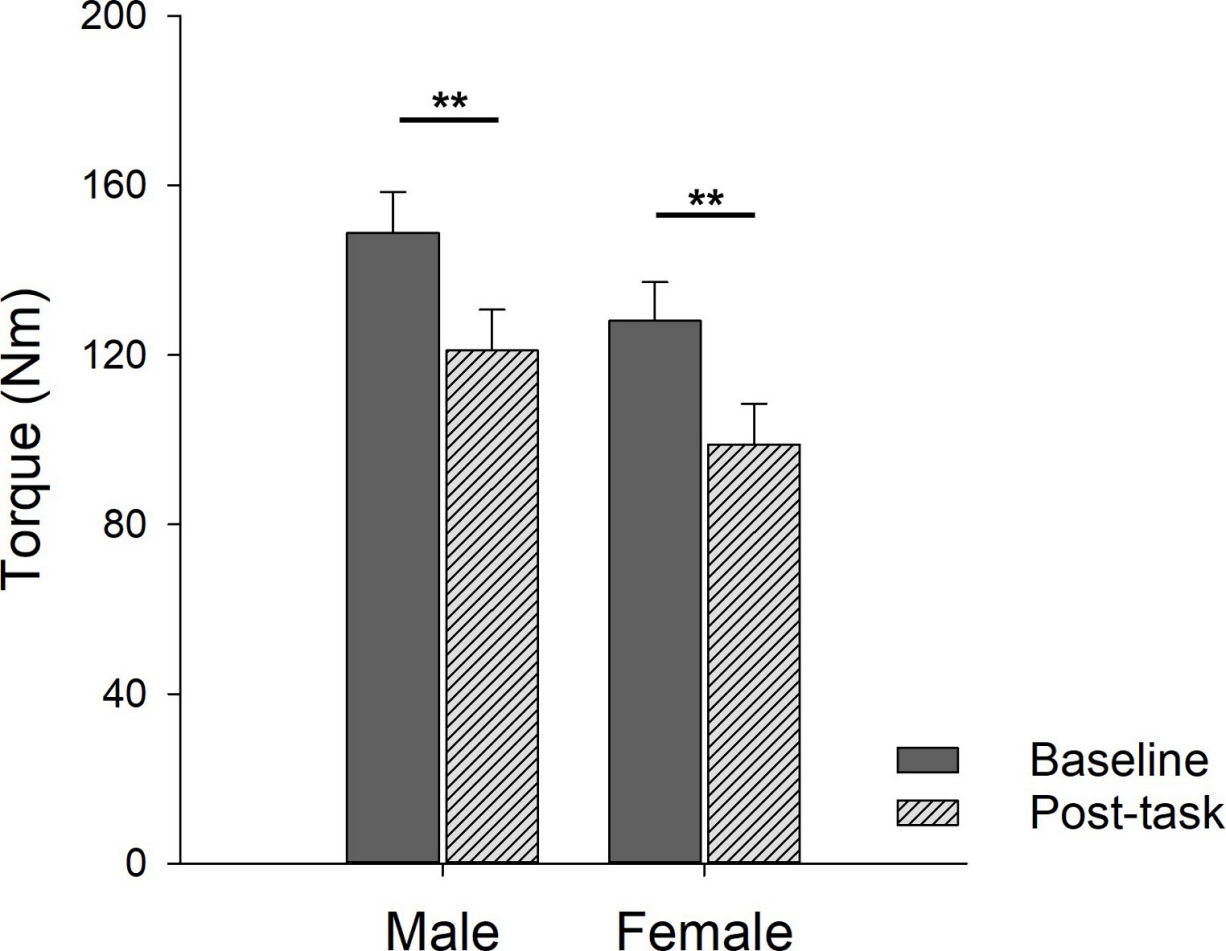
MVIC torque before and after the SLHR in males and females. Torque was reduced following SLHR task for both males (dark gray, 18.6% reduction from baseline) and females (light gray, 22.7% reduction from baseline). ** p<0.01.

There was no difference in the number of SLHR repetitions performed to task failure by males and females (p = 0.14, Fig 2). At baseline, there was no sex differences in MVIC torque (p = 0.198, Fig 3, “baseline”). The SLHR task resulted in 20.5% reductions in MVIC torque from baseline for all participants (time effect, p<0.001). However, there was no sex difference in the reduction of MVIC (sex effect, p = 0.14, males 18.6% and females 22.7%; Fig 3) and no interaction of sex and time (p = 0.865). Normalizing MVIC torque to body weight did not change these results: baseline torque remained similar between groups (*p* = 0.479); MVIC torque following the SLHR was not different between groups (*p =* 0.808); and post-task reduction in MVIC was similar between males and females (*p =* 0.532). Furthermore, body weight did not mediate the relationship between sex and repetitions completed during the SLHR (*p* = 0.206).

### EMG

**Fig 4:**
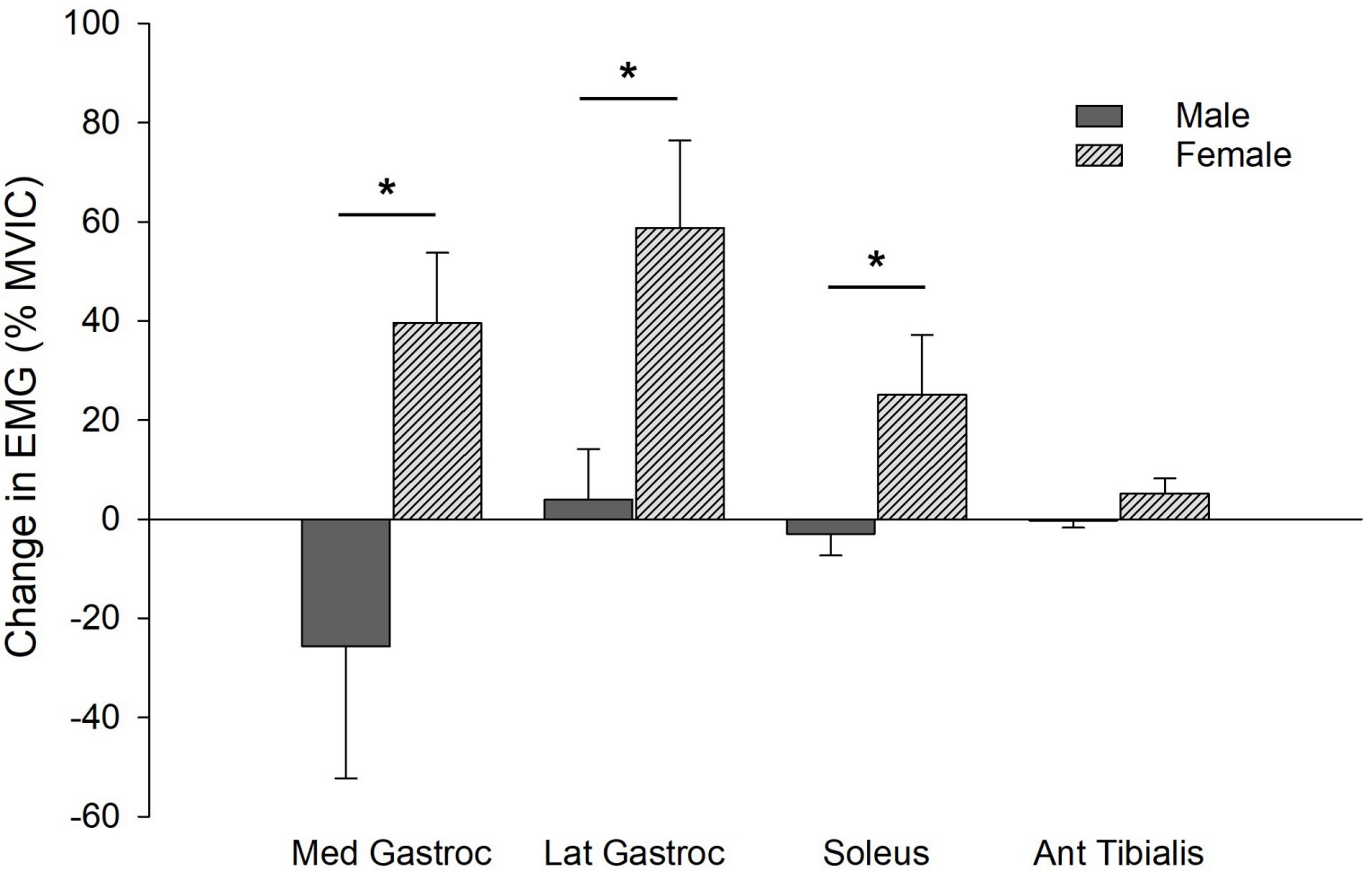
Change in RMS EMG amplitude for each the soleus, medial and lateral gastrocnemius, and tibialis anterior muscles from the start to end of the SLHR task, as a percent of baseline MVIC. EMG for all three plantar flexor muscles increased in females (striped bars). In males (solid bars), medial gastrocnemius EMG decreased, while lateral gastrocnemius and soleus EMG remained unchanged. Tibialis anterior EMG remained unchanged in both males and females. *, interaction between sex and time, *p*<0.05.

RMS EMG (% MVIC) amplitude of the triceps surae did not change from the start to the end of the SLHR task for males (MG: 17.3% decrease, *p =* 0.351; LG: 12.2% increase, *p =* 0.081; SOL: 1.9% decrease, *p =* 0.683), but females had significant increases in all three muscles (sex and time interaction for combined triceps surae muscles, *p* = 0.029). The females demonstrated increases in the MG (32.0% increase, *p =* 0.026), LG (52.3%, *p =* 0.011) and SOL (26.4%, *p =* 0.030) (Fig 4). Bodyweight did not mediate the sex-differences in EMG for any of the triceps surae muscles (MG, *p =* 0.176; LG, *p =* 0.375; SOL, *p =* 0.070).

### Voluntary activation and twitch amplitude

**Fig 5:**
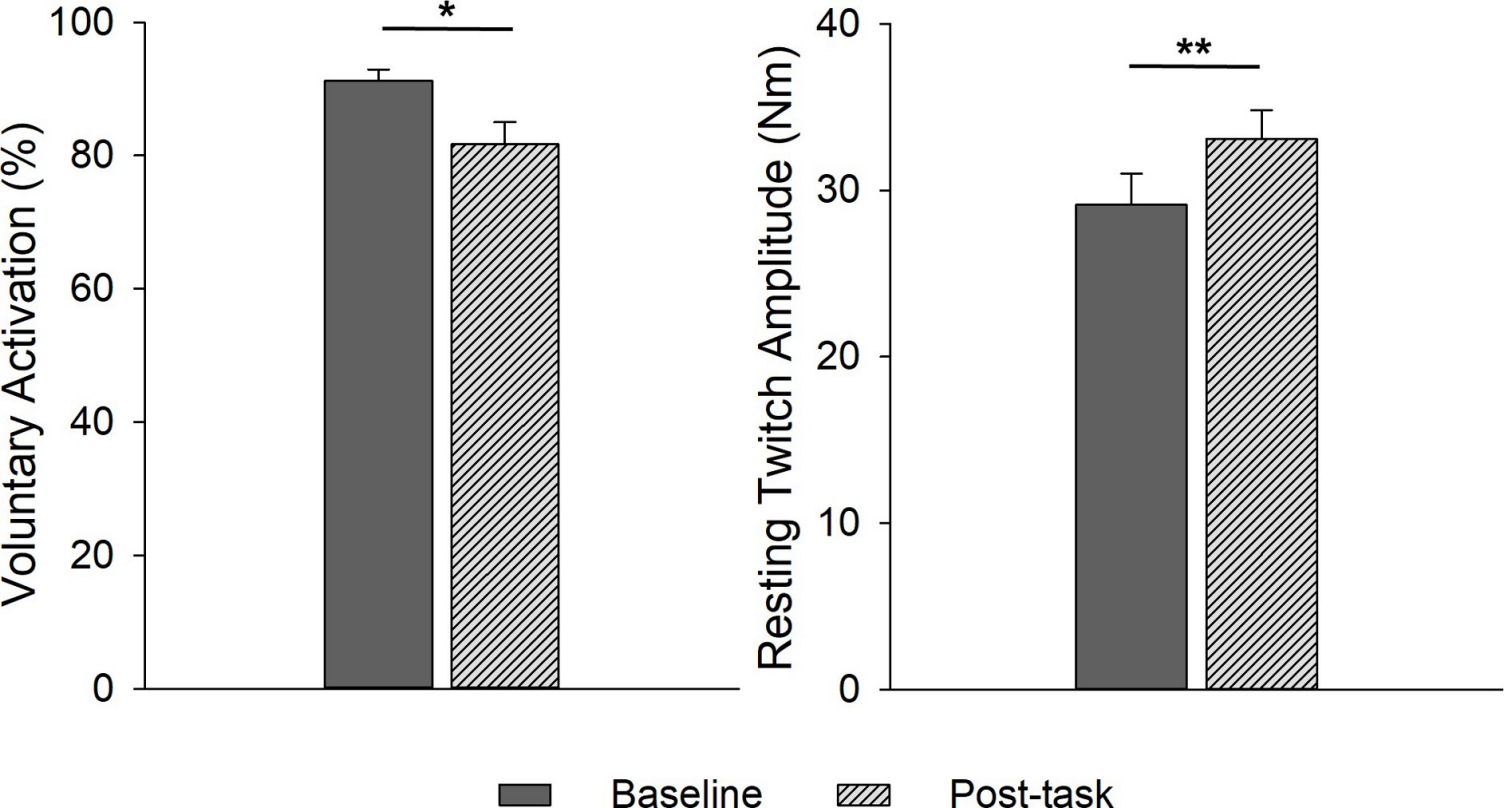
Voluntary activation (A) and resting twitch amplitude (B) before and after the SLHR task in males and females pooled. Voluntary activation decreased (p = 0.01) and resting twitch amplitude increased (p<0.001) following the SLHR task for males and females (there were no sex differences in response to the SLHR). * *p*<0.05; ** p<0.01.

There were no sex differences in baseline voluntary activation (VA, p = 0.866) or post-task reduction in VA (p = 0.856). There was a significant main effect of time: the SLHR resulted in reduced VA for all participants (9.8% decrease from baseline, p = 0.012; Fig 5). There were similarly no sex differences in either baseline resting twitch amplitudes (RT, p = 0.057) or post-task change in RT (p = 0.357, Fig 5). There was a significant main effect of time: RT increased from pre- to post-task for all participants (17.6% increase from baseline, p<0.001, Fig 5).

### Associations

The number of SLHR repetitions performed was not correlated with either the baseline MVIC (r = -0.005, p = 0.979) or the percent reduction in MVIC following the SLHR (r = -0.234, p = 0.23). The percent reduction in MVIC was positively associated with the reduction in VA (r = 0.841, p<0.001): those who had the greatest reductions in MVIC also had the largest reductions in VA. However, the SLHR was not associated with either baseline VA (r = 0.354, p = 0.064), reduction in VA (r = -0.085, p = 0.669), baseline RT (r = 0.054, p = 0.786), or the change in RT (r = -0.104, p = 0.597). Baseline MVIC was not predictive of change in MVIC (r = 0.122, p = 0.537): those with greater MVIC torque did not necessarily have greater (or lesser) reductions in MVIC torque following the SLHR.

## Discussion

The novel findings of this study were: (1) the SLHR (number of repetitions completed at task failure) was not associated with the maximal isometric plantar flexion strength (MVIC) nor the relative reduction in maximal strength after the SLHR (fatigability of the MVIC), and (2) there were no sex differences in either SLHR repetitions completed, MVIC and fatigability of the MVIC, or associations between these variables. Additionally, our results implicate reductions in voluntary activation (i.e., neural drive) as a primary mechanism for fatigability of the MVIC after SLHR in both males and females. Collectively, our results suggest that the SLHR is not a valid measure of strength but a specific measure of dynamic-contraction lower limb fatigability that is similar in males and females.

### Fatigability and strength

The number of dynamic SLHR repetitions were not correlated with maximal isometric plantar flexion strength. From a physiological standpoint, this is expected; strength, represented in this study by brief maximal isometric voluntary efforts, is typically correlated most strongly with physiological cross-sectional area, which is indicative of the number of sarcomeres in parallel in a muscle fiber and muscle [22]. Strength is often not predicted by the ability to maintain a task for a period of time because the physiological mechanisms involved in continued task performance are quite different from those involved in generating maximal force for a brief time [7]. Specifically, failure to maintain the requirements of task over a period of time (in this case, ~100 seconds) involves the ability of the muscle to meet the metabolic demands based on the task requirements. During dynamic fatiguing contractions, fiber velocity slows and maximal force development declines due to increased metabolites within the muscle (e.g. inorganic phosphate, hydrogen ions), thereby resulting in fatigability [23,24]. Furthermore, afferent feedback (group III and IV) due to increased metabolite accumulations can inhibit motor neurons in the spinal cord and reduce voluntary drive [9,25]. Thus, the physiological mechanisms most relevant to the SLHR are the ability of the muscle to meet the metabolic demands of the fatiguing task and the ability to maintain neural drive to the muscle, not the physiological cross-sectional area of the muscle.

In the clinical setting, the SLHR is thought to measure plantar flexion strength, as purported in manual muscle testing procedures [1]. However, combined with the absence of physiological basis, the lack of association between the SLHR and maximal plantar flexion strength in young, healthy participants calls into question the construct validity of the SLHR. Importantly, our finding agrees with a prior study evaluating 43 adults with myositis (64.9 years old), where SLHR repetitions were not correlated with plantar flexion MVIC [5]. Concerns with the SLHR have arisen previously, including: lack of uniform testing parameters [2,26]; and limited sensitivity (specifically, an inability to differentiate outcomes between treatment groups) [27]. Therefore, whether for healthy or clinical populations, the SLHR is likely an invalid measure of maximal plantar flexion strength.

The plantar flexors experienced reductions in MVIC following the SLHR. However, these reductions in force were not associated with performance in the SLHR. In single-joint tasks performed to failure, maximal strength or power is expected to decrease following task failure [24]. This lack of association between the SLHR and reduction in MVIC indicates that fatigability of the plantar flexors is task-dependent [10], as seen in other lower limb muscle groups. For example, in young, healthy adults, the fatigability of the knee extensor muscles and involved mechanisms differed for an isometric fatiguing contraction compared with a dynamic knee extensor tasks [14] because the specific demands of the task dictate the amount of fatigue and the mechanisms. A decrease in MVIC (isometric) after the SLHR may reflect a reduction in the number of active cross-bridges [24,28] and, as we showed, a reduction in neural drive (see below). In contrast, a slowing of velocity during the dynamic contraction fatigue of a SLHR likely reflects the limits of crossbridge speed and reduction in maximal shortening velocity of the fiber [29].

Importantly, the SLHR may not be limited to the ankle plantar flexor muscles. Apart from fingertip support for balance, there are no methods used to stabilize the body or surrounding joints during the SLHR. Thus, it is probable that muscles external to the plantar flexors–including foot intrinsic and extrinsic musculature [30,31], knee extensors, frontal plane hip stabilizers, and core musculature–are additionally responsible for success (and ultimately failure) in the SLHR. The SLHR may, therefore, be best interpreted as a functional, dynamic task evaluating fatigability of the entire lower extremity.

### Mechanisms

Our study evaluated measures representing neural and contractile mechanisms that may contribute to the reduction in MVIC strength of the plantar flexor muscles after the SLHR and variability in performance of the SLHR. Voluntary activation–assessed during the MVIC with electrically-evoked contractions–was reduced following the SLHR and was correlated with the reduction in MVIC torque, suggesting that decreased neural drive contributed to fatigability of the MVICs. Conversely, changes in resting twitch amplitude were not related to the reduction in MVIC, suggesting that contractile mechanisms within the plantar flexor muscle group were not responsible for the loss in maximal plantar flexion force following the SLHR. This is atypical for dynamic tasks that are constrained to a single limb; typically, changes in maximal strength following dynamic fatiguing exercise are explained by contractile mechanisms, as in the knee extensor muscles [14]. In the case of the SLHR, this difference may highlight the whole-limb nature of this task. Without constraining neighboring joints and providing robust balance assistance, several other muscles must be innervated to enable task continuation. As a result, the burden on the central nervous system may be greater. Thus, while the SLHR fails to predict either maximal plantar flexion strength or the magnitude of the reduction in MVIC, perhaps it is best interpreted as an assessment of whole lower limb dynamic fatigability.

There were no sex differences in SLHR repetitions completed before task failure, nor were there differences in maximal strength or fatigability. The lack of sex differences in strength was surprising given that, on average, men are stronger than women—although more so for upper limb muscles [32]. There were additionally no sex differences in the mechanisms driving fatigability in the SLHR, including the reduction in voluntary activation, which was similar in males and females. Neural mechanisms (i.e., voluntary activation) were similarly associated with fatigability in males and females, while contractile mechanisms (i.e., resting twitch amplitude) bore no relationship to fatigability in either males or females.

Sex differences were observed only in muscle activation levels (EMG amplitudes) during the SLHR. While males had no increase in EMG amplitudes, females had significant increases for each the soleus, medial and lateral gastrocnemius muscles. Typically, repeated submaximal contractions result in progressive increases in EMG [6], representative of further recruitment of motor units as muscle fibers become progressively fatigued [33]. This was evident in the females, but it was not observed in the males, suggesting that, at least for females, the SLHR was a submaximal, fatiguing task. The relatively stable soleus and lateral gastrocnemius EMG amplitudes and the reduction in medial gastrocnemius EMG amplitude seen in male participants could indicate that the SLHR was a maximal task for their plantar flexors. However, when combined with the knowledge that their voluntary activation and MVIC torque both reduced–and to a similar magnitude as female participants–there is one explanation that seems more plausible: although the rate of fatigue of the dynamic contractions was similar for the males and females, they fatigued for different reasons. The EMG data indicate that males and females utilized different strategies and recruitment of muscles during the SLHR.

SLHR performance is correlated with many measures of functional relevance: joint stability, proprioception and balance [34,35]; gait speed and use of an assistive device [36–38]. This makes understanding the fatigability of the plantar flexors and surrounding, supporting musculature during the SLHR even more critical. For instance, strengthening of intrinsic foot musculature alone improved heel raise height and repetitions completed in persons with flat feet [31]. This finding supports our conclusion that SLHR performance depends upon more than just plantar flexor function, and it suggests that neuromuscular mechanisms should be investigated as they relate to fatigability of intrinsic foot muscles. To date, however, the degree to which intrinsic foot muscles contribute to SLHR performance has not been investigated, nor have the contributing roles of other muscles acting at the foot, knee, or hip joints.

There were no sex differences in SLHR repetitions completed in this study, even when accounting for differences in body weight. Total work performed, however, may have differed between males and females as a function of ankle range of motion [3]. Furthermore, differences in muscle function proximal and distal to the talocrural joint may impact task performance between males and females. The role of stabilizing muscles in the SLHR, including the neuromuscular mechanisms of these muscles that contribute to task failure, would assist in identifying whether further sex differences in SLHR task performance exist and whether these need to be assessed and addressed during clinical evaluation.

### Limitations

Maximal isometric voluntary contractions were used to assess fatigability after the SLHR, despite the dynamic nature of the task. This selection was intentional because: (1) manual muscle testing (MMT) is traditionally performed as an evaluation of maximal *isometric* strength; and (2) assessment of voluntary activation during maximal dynamic contractions in this setting is not yet a reliable technique. Furthermore, there was ~45 seconds of delay in measuring post-task maximal strength and contractile properties assessed in the dynamometer. Any pre- to post-task differences, therefore, reflect the time of measurement and were not precisely immediately post-task. Finally, lack of a familiarization session could have impacted MVIC torque [9]. However, it is unlikely that the groups would have been differentially impacted by the lack of a familiarization session. Furthermore, young to middle-aged adults do not exhibit sex differences in activation [18,39] and typically require minimal practice to achieve optimal activation in a session with no prior familiarization [40].

Testing in the Biodex utilized a flexed-knee position, while the SLHR utilizes a straight-knee position. This could affect interpretations of relative muscle contributions in the SLHR when compared to MVICs. However, no such comparisons were made between tasks. Importantly, while the change in knee position may result in altered PF torque, this would not impact the relationship between the SLHR and MVIC torque: the approximate 85% reduction in PF torque that occurs from 0 degrees to 90 degrees knee flexion [41] would simply be as though a constant multiplier were added to the equation evaluating correlation between SLHR reps and MVIC torque, which would not impact the detection of a relationship between these variables. Finally, neither sex, BMI category, nor physical activity level impact the relationship between ankle angle and peak PF muscle activity [42]. Thus, the choice of testing position in the Biodex is unlikely to have impacted the between-group findings in this study.

## Conclusions

The SLHR is a poor predictor of maximal plantar flexor strength in young, healthy males and females. As a test of repetitions to failure, the SLHR is well-suited to evaluate performance fatigability. However, as a task whose performance is dependent upon several factors—including coordination and stabilization of proximal and distal muscles and joints—the SLHR more likely evaluates dynamic fatigability of the lower extremity. Additionally, there were no sex differences in the number of SLHR repetitions, nor were there sex differences in maximal baseline plantar flexor strength or plantar flexor fatigability. Based on differences in muscle activation patterns, males and females likely utilize different strategies to succeed in the SLHR.

### Clinical relevance

The results of this study reveal a critical limitation in current interpretation of the SLHR: the lack of association between SLHR repetitions and MVIC diminishes the validity of using the SLHR as a clinic-based evaluation of plantar flexor strength. Furthermore, the lack of relationship between SLHR repetitions and reduction in plantar flexion MVIC suggests that the cause of task failure of the SLHR may not be specific to the plantar flexor muscles. Instead, this task may be best interpreted as a task of dynamic lower extremity fatigability rather than strength or fatigability specific to the primary plantar flexor muscles involved in the task. Modifications have previously been suggested to improve the psychometric properties of the SLHR, including construction of various specialized devices, addition of linear encoders, utilization of a metronome, and addition of a weight belt [26,43–45]. However, the same limitations exist for these modified protocols: number of repetitions to failure cannot predict plantar flexor strength, and the SLHR task is not specific to the plantar flexor muscles. Thus, to evaluate maximal plantar flexor strength in clinic, new clinical measures need to be developed.

## Supporting information

S1 Dataset (XLSX)
